# Progress in Access and Oral Polio Vaccine Coverage Among Children Aged <5 Years in Polio Campaigns After the Political Change in Afghanistan

**DOI:** 10.1093/infdis/jiae129

**Published:** 2024-04-10

**Authors:** Wrishmeen Sabawoon, Shion Seino, Bakht Mohmmad Pason, Nek Wali Shah Momin, Sayako Kanamori, Connor Bender, Kazuhisa Takemura

**Affiliations:** General Incorporated Association LIAISON, Yokohama, Kanagawa, Japan; General Incorporated Association LIAISON, Yokohama, Kanagawa, Japan; National Emergency Operational Center, Ministry of Public Health, Kabul City, Kabul Province, Afghanistan; National Emergency Operational Center, Ministry of Public Health, Kabul City, Kabul Province, Afghanistan; Center for Education in Liberal Arts and Sciences, Osaka University,, Osaka; General Incorporated Association LIAISON, Yokohama, Kanagawa, Japan; Center for Decision-Making Research, Waseda University, Shinjuku-ku, Tokyo, Japan

**Keywords:** inaccessibility, oral polio vaccine coverage, surveillance, poliovirus, Afghanistan

## Abstract

**Background:**

Warfare has long impeded vaccination programs in polio-endemic Afghanistan. We aimed to describe progress in access to children under 5, oral polio vaccine (OPV) coverage among children under 5 in nationwide polio campaigns, and polio surveillance performance indicators after the Islamic Republic of Afghanistan collapsed to Taliban forces in August 2021.

**Methods:**

Trends in the number of wild poliovirus type 1 (WPV1) and circulating vaccine-derived poliovirus type 2 (cVDPV2) cases and surveillance indicators from 2015 to 2023, and trends in the OPV coverage in the November 2020–June 2022 polio campaigns, were described.

**Results:**

From 2015 to mid-July 2020, 74 of 126 (58.7%) WPV1 cases were reported from inaccessible areas. In November 2020, 34.1% of target children under 5 were inaccessible; in November 2021 (the first postchange polio campaign), all were accessible. From November 2020, under-5 OPV coverage of 69.9% rose steadily to 99.9% in the May 2022 campaign. The number of cVDPV cases fell from 308 (2020) to zero (2022). June 2022's house-to-house OPV coverage was 34.2% higher than non–house-to-house modalities. Nonpolio acute flaccid paralysis and stool adequacy rates rose from 18.5/100 000 and 92.6% in 2020 to 24.3/100 000 and 94.4% in 2022, respectively.

**Conclusions:**

Children's inaccessibility no longer vitiates polio eradication; polio surveillance systems are less likely to miss any poliovirus circulation.

## Teaser

After the August 2021 political change in Afghanistan, millions of inaccessible target children in polio campaigns became accessible. The change improved oral polio vaccine coverage and polio surveillance performance. Nonhousehold implementation of polio campaigns remains a significant challenge.

## Graphical Abstract

**Graphical Abstract:**
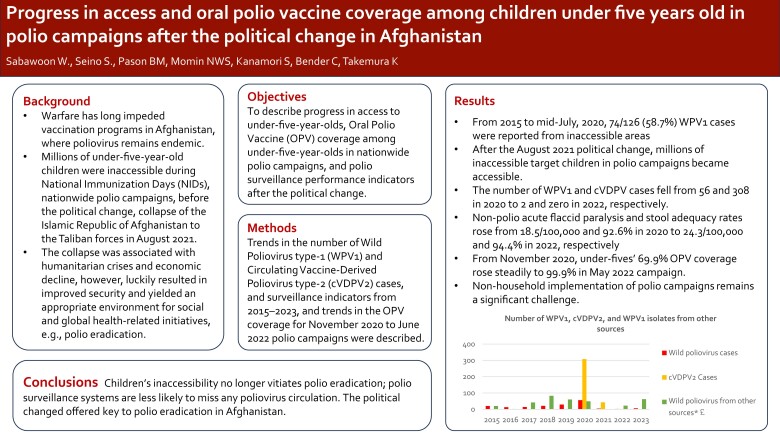

Polio is a highly infectious disease caused by 3 types of wild poliovirus (WPV). Based on successful polio control efforts in the Americas, the World Health Organization (WHO) launched the Global Polio Eradication Initiative (GPEI) in 1988, one of human history's largest-ever public health interventions [1, 2]. Thanks to GPEI efforts, WPV cases went from an estimated 350 000 in 1988 in >125 countries to 174 in 2019; in 2021, with no more than 6 cases in total, Afghanistan (4), Pakistan (1), and Malawi (1) recorded fewer WPV cases than ever before. In 2022, however, the number of cases rose to 30: Afghanistan (2), Pakistan (20), and Mozambique (8). In 2023 (as of 31 December 2023), only 12 polio cases have been reported, in Afghanistan (6) and Pakistan (6). The oral polio vaccine (OPV) has enabled the GPEI to successfully eradicate the virus from all major parts of the world except Pakistan and Afghanistan, where it remains endemic [3, 4]. However, circulating vaccine-derived polioviruses (cVDPVs) occur: Where eradication efforts fail, viruses reacquire neurovirulence after prolonged circulation in seriously underimmunized communities. Eradication of all WPV type 1 (WPV1) and cVDPV variants is now the highest priority for GPEI. Following the switch from trivalent OPV to bivalent OPV, the type 2 OPV (OPV2) global withdrawal from vaccination in April 2016, outbreaks have occurred in several countries. In 2020 and 2023, there were 1117 (96.9% VDPV2) and 473 (72.5% cVDPV2) cVDPV cases, respectively. Due to warfare and deteriorating security, poor routine immunization, and programming challenges, Afghanistan also experienced a serious cVDPV2 outbreak from 2020 (308 cases) to 2021 (43 cases) [5].

Afghanistan has long suffered from civil and international wars, including the war between the Afghan government and the USSR’s (Union of Soviet Socialist Republics) allies with the Mujahideen, interfactional warfare between Mujahideen and the Taliban, and war between the Taliban and the Afghan government and its North Atlantic Treaty Organization (NATO) allies [6–8]. The latest war continued for almost 2 decades and resulted in the collapse of the NATO-backed government to the Taliban in August 2021.

The political change has been associated with economic deprivation, humanitarian crises, and disruption of services, including healthcare [9]. For decades, these wars impaired or prevented economic development, affected the health of the population [7], resulted in massive internal displacements [7–10] such as the emigration of millions of refugees to Pakistan and Iran [7], and caused millions of casualties [7, 8]. Despite tremendous efforts to reconstruct the healthcare system [11, 12], warfare has long impeded routine immunization coverage [13] and the implementation of polio campaigns in Afghanistan [14, 15]. More specifically, armed conflict has resulted in the underimmunization of the population in certain areas and children's inaccessibility during polio vaccination campaigns. These factors, together with the suboptimal quality of the polio campaigns in accessible areas and insufficient routine immunization programs, resulted in a continuous immunity gap against polio [14, 16–19].

Previous studies have indicated that countries with polio cases have high rates of infant mortality, lower levels of peace, more displaced populations, higher violent crime rates, and higher political instability [20]. In addition to geographic location, sex, and socioeconomic status, which are known factors affecting access to immunization services [21], conflict conditions also determine lower immunization rates and spread of poliovirus in low- and/or middle-income countries. In Pakistan, fighting between the government's military forces and insurgents has proliferated WPV [22]. Here, we describe how inaccessibility due to security threats imperils polio eradication, and report significant rapid progress in the acute flaccid paralysis (AFP) surveillance indicators and in the OPV coverage of children aged <5 years (hereafter “under-5s”) in polio campaigns conducted after inaccessibility was resolved following Afghanistan's political transition (hereafter “change”).

## METHODS

We obtained data on national immunization days, the countrywide polio campaigns for the years 2020 to 2022, from the National Emergency Operations Center, Ministry of Public Health (MoPH), Afghanistan. Data on AFP, WPV1, and cVDPV2 cases and the polio surveillance performance indicators for the years 2015 to 2023 were obtained from WHO's Eastern Mediterranean Regional Office and GPEI websites [4, 5, 23]. To assess progress in the performance of AFP and environmental surveillance, we described trends in the number of AFP, WPV1, and cVDPV2 cases, AFP surveillance performance indicators, and sewage sampling sites from 2015 to 2023. To describe how inaccessibility affects polio occurrence, we described the trend in the proportion of WPV1 cases reported from inaccessible areas. To assess progress in access to children and OPV coverage among children under 5, we described trends in the numbers and rates of vaccinated, inaccessible, and missed children and refusals in polio campaigns conducted postchange to visualize progress compared to polio campaigns conducted prechange, November 2020 and January 2021. To differentiate the OPV coverage between house-to-house and non–house-to-house implementation modalities, we described trends in the numbers and rates of children vaccinated by the various modalities of supplementary immunization activities (SIAs). Details on background information, access dynamics’ impacts on the implementation of polio campaigns, and methodology are available in the Supplementary Annex.

## RESULTS

### Number of AFP, WPV1, and cVDPV2 Cases, 2015–2023

WPV1 cases increased from 20 in 2015 to 56 in 2020. Postchange, there were 2 in 2022 and 6 in 2023. Prechange (1 January 2015 to 13 July 2020), a total of 126 WPV1 cases had been reported, 74 (58.7%) from inaccessible areas and 52 (41.3%) from accessible areas. The proportion of WPV1 cases reported from inaccessible areas increased from 10 of 20 (50.0%) in 2015 to 24 of 29 (82.8%) by 13 July 2020. The huge outbreak of cVDPV2 in 2020 with 308 cases decreased to 43 cases in 2021. Fortunately, there being zero postchange cases means circulation has stopped (Table 1).

**Table 1.** Number of Acute Flaccid Paralysis (AFP), Wild Poliovirus Type 1, and Circulating Vaccine-Derived Poliovirus Type 2 Cases, Nonpolio AFP Rate, Percentage With Adequate Stool Specimens, and Sewage Sampling Sites, 2015–2023

Year	AFP Cases, No.	Annualized Nonpolio AFP Rate	% With Adequate Stool Specimens	WPV1 Cases			WPV From Other Sources^a,b^	cVDPV2 Cases, No.	Environmental Sampling Sites, No.^c^
				Total, No.	Accessible, No. (%)	Inaccessible, No. (%)			
2015	2738	13.8	93	20	10 (50.00)	10 (50.00)	20	0	14
2016	2905	14.4	92.2	13	4 (30.77)	9 (69.23)	2	0	15
2017	3094	15.2	93.5	14	2 (14.29)	12 (85.71)	42	0	20
2018	3378	16.6	93.8	21	10 (47.62)	11 (52.38)	83	0	20
2019	3768	18.1	94.1	29	24 (82.76)	5 (17.24)	60	0	21
2020	3972	18.5	92.6	56	24 (82.76)^d^	5 (17.24)^d^	49	308	23
2021^e^	4088	18.9	93.5	4	NA	NA	1	43	26
2022	5368	24.34	94.4	2	NA	NA	22	0	32
2023	5842	25.9	94.2	6	NA	NA	62	0	38

Data sources: World Health Organization Eastern Mediterranean Regional Office [23], Global Polio Eradication Initiative [4, 5], and National Emergency Operational Center of the Ministry of Public Health.

Abbreviations: AFP, acute flaccid paralysis; cVDPV2, circulating vaccine-derived poliovirus type 2; NA, not applicable; WPV, wild poliovirus; WPV1, wild poliovirus type 1.

^a^WPV1-positive isolates from environmental samples, selected contacts, and healthy children.

^b^Other sources include sewage samples, close contacts of the polio case, and stool specimens collected from healthy children. The majority comes from sewage sample (environmental sample) collection sites which were located in accessible areas before the political change.

^c^Sewage sampling site for the detection of poliovirus: 2 sites in 2013 and 40 sites in 2024.

^d^Data are from January to 13 July 2020.

^e^The political change occurred in August 2021.

### AFP Surveillance Performance Indicators and Environmental Sites for the Detection of Poliovirus, 2015–2023

Reported AFP cases increased from 2738 in 2015 to 3972 in 2020. Postchange, 5368 were reported in 2022 and 5842 in 2023. Standard performance indicators also improved postchange (Table 1). Regarding environmental surveillance, the number of sewage collection sites, 2 in 2013, gradually increased to 23 sites in 2020. Postchange, the number increased to 38 in 2023 and 40 in 2024.

### Number of Inaccessible Children

There had been no campaign without access issues in the past 2 decades. Access to target children deteriorated and became more complicated after 2016 (see Supplementary Annex): a total of 413 717 target under-5s were inaccessible in October 2016, 107 482 in September 2017, 1 311 011 in August 2018, 4 921 081 in August 2019, and 3 408 293 in November 2020. In November 2021, however, inaccessible target children's numbers fell to zero when the Islamic Emirate of Afghanistan (Taliban) conducted its first campaign and the MoPH and GPEI partners conducted successful SIAs from November 2021 to June 2022 and onward, as the program now had access to all children throughout Afghanistan (Table 2).

**Table 2.** Trend in the Number of Vaccinated, Inaccessible, and Missed Children and Refusals and Their Rates by Round and Implementation Modality of Polio Campaigns

Target	Reference NIDs		Campaigns Conducted Postchange						
	Nov 2020	Jan 2021	Nov 2021	Dec 2021	Jan 2022	Feb 2022	Mar 2022	May 2022	Jun 2022
Target under-5s	9 999 227	9 270 597	9 999 227	9 806 328	9 366 998	6 375 723	9 643 304	9 999 227	9 969 284
Target under-5s in cold districts	46 301	728 630	None	192 899	632 229	719 906	355 923	None	None
Inaccessible target under-5s	3 408 293 (34.1)	3 086 735 (33.3)	0	0	0	0	0	0	0
Total coverage (vaccinated target children)	6 990 755 (69.9)	6 599 250 (71.2)	8 556 783 (85.6)	8 695 580 (88.7)	8 294 824 (88.6)	6 347 320 (99.6)	9 460 653 (98.1)	9 993 058 (99.9)	10 061 780 (100.9)
Coverage by NIDs implementation modality									
House-to-house									
Target	6 158 542	5 897 926	5 283 681	5 534 440	4 965 924	5 101 303	6 207 255	7 180 055	7 180 055
Vaccinated	5 657 867 (91.9)	5 368 023 (91)	5 375 177 (101.7)	5 663 432 (102.3)	5 179 313 (104.3)	5 407 742 (106)	6 676 471 (107.6)	7 783 746 (108.4)	7 835 507 (109.1)
Mosque-to-mosque									
Target	NA	NA	4 645 478	4 271 888	4 142 042	913 742	2 019 303	1 255 913	1 255 913
Vaccinated	NA	NA	3 119 663 (67.2)	3 032 148 (71)	2 951 361 (71.3)	596 840 (65.3)	1 687 461 (83.6)	935 335 (74.5)	884 262 (70.4)
Site-to-site									
Target	1 012 514	840 246	NA	NA	NA	NA	1 071 226	793 636	763 693
Vaccinated	185 598 (18.3)	166 385 (19.8)	NA	NA	NA	NA	939 728 (87.7)	612 408 (77.2)	607 506 (79.5)
Mixed									
Target	1 813 220	1 613 299	70 068	NA	225 848	360 678	345 520	769 623	769 623
Vaccinated	1 147 290 (63.3)	1 064 842 (66)	61 943 (88.4)	NA	164 150 (72.7)	342 738 (95)	156 993 (45.4)	661 569 (86)	734 505 (95.4)
Total recorded missed children (absent, neonatal, sleeping, sick) in the house-to-house campaign	146 846 (2.4)	140 140 (2.4)	134 733 (2.5)	138 948 (2.5)	123 790 (2.5)	142 315 (2.8)	152 779 (2.5)	152 035 (2.1)	131 202 (1.8)
Recorded missed due to refusal of parents in the house-to-house campaign	58 418 (0.9)	57 530 (1)	30 807 (0.6)	29 407 (0.5)	32 377 (0.7)	39 158 (0.8)	39 332 (0.6)	36 136 (0.5)	40 140 (0.6)

Data are presented as No. or No. (%).

Abbreviations: NA, not applicable; NID, national immunization day (massive countrywide polio campaign).

### Under-5 OPV Coverage, Number of Missed Children and Refusals, and Their Rates

The number of target under-5s in Afghanistan is estimated to be slightly less than 10 million; the number varies among different types of SIAs and by areas to be covered. OPV coverage among under-5s was 6 990 755 (rate 69.9%) in November 2020 and 6 599 250 (71.2%) in January 2021. Coverage progressively increased through postchange SIAs, ranging from 8 581 895 (85.8%) in November 2021 to 9 460 653 (98.1%) in March 2021 and 10 061 780 (100.9%) in June 2022 (note that the rolling denominator is a polio campaign estimate, as no census has been conducted in Afghanistan for 60 years). Recorded missed children shrank from 146 846 (2.4%) in November 2020 and 140 140 (2.4%) in January 2021 to 131 202 (1.8%) in June 2022. Target under-5s’ OPV refusals in house-to-house campaign areas also declined from 58 418 (0.9%) in November 2020 and 57 530 (1.0%) in January 2021 to 36 136 (0.5%) in May 2022 and 40 140 (0.6%) in June (Table 2).

### Under-5 OPV Coverage by Polio Campaign Modality

Prechange, the most recent polio campaigns were conducted with a mixed approach—house-to-house in areas under the control of the former government, but mainly site-to-site in areas formerly under the control of the Taliban. Postchange, all SIAs were house-to-house in formerly government-controlled areas, and mosque-to-mosque, site-to-site, or mixed in other settings mainly under the prechange influence or control of the Taliban. The percentage of target children vaccinated in house-to-house campaigns increased from 91.0% in January 2021 to >100.0% in all postchange SIAs. Under-5 OPV coverage was still far lower in both mosque-to-mosque and site-to-site polio campaigns than in house-to-house visits postchange (Table 2).

## DISCUSSION

This study reports significant progress in access to every child, in polio surveillance, and in overall OPV coverage in Afghanistan required for the final push to global polio eradication. It also identifies shortcomings in the implementation of the polio campaigns that should be promptly addressed by the GPEI.

### Progress in Access and OPV Coverage

This study quantified the impacts of inaccessibility to children in Afghanistan by identifying trends in reported numbers of WPV1 cases, comparing accessible and inaccessible areas; it assessed progress via trends in numbers of vaccinated, inaccessible, and missed children and refusals, and their rates among target under-5s pre- and postchange in Afghanistan.

On average over a 5-year period, 58.7% of the 125 WPV1 cases were reported from inaccessible areas. The percentage increased from 50.0% (10/20) of cases in 2015 to 82.8% (24/29) by mid-July 2020. The findings are concordant with studies that reported associations of insecurity with an increased incidence rate of polio [24] and of measures of violence and instability with polio incidence [25]. Earlier studies and progress reports on polio eradication initiatives did not stratify polio incidence by access status of areas where polio cases occurred [14, 16–19]. This study's stratification quantified the impact of inaccessibility on polio eradication. The progressive increase in the number of WPV1 cases from inaccessible areas highlights the significance of inaccessibility for polio eradication in 1 of the last 2 endemic countries. In contrast to the percentage of cases reported from inaccessible areas, 41.7% of WPV1 cases were reported from accessible areas, indicating that immunity gaps can also be a consequence of poor routine immunization programs and suboptimal polio campaigns in certain accessible areas [14, 26, 27].

Inaccessibility to children during SIAs due to conflict conditions in prechange Afghanistan was long a key challenge to global polio eradication. Based on US Pentagon Defense Intelligence data, the number of Taliban-initiated attacks on government forces and/or its NATO allies increased from 372 in 2002 to 31 879 in 2010 and 40 536 in 2020 [8]; although presumably there might be a comparable number of government and NATO-initiated attacks on the Taliban, their exact number is unknown to us. The political and military situations were complex and inaccessibility to children had both a constant and a dynamic nature: some areas were persistently inaccessible and some areas were accessible in 1 round of an SIA but inaccessible in the next. Some areas were fully inaccessible; some areas were partially accessible; some areas were accessible with constraints (eg, program staff monitoring was forbidden, and/or SIA staff selection was done by the Taliban rather than by the program) while some areas under government control were fully accessible [28]. During the study period, the Taliban imposed on-and-off bans on the polio campaigns in areas under their control or at the national level. This volatility resulted in the nonvaccination of hundreds of thousands to millions of target under-5s or nonvaccination during several rounds at the national level: the number of inaccessible children, 413 717 under-5s in October 2016, rose to 4 921 081 in August 2019, and was 3 408 179 in November 2020 and 3 086 735 in January 2021 (Table 2, Supplementary Annex).

The Taliban leadership was not categorically opposed to polio vaccination, though they modified campaign modalities in some areas to thwart the government's and government allies’ intelligence gathering in areas under Taliban control, or to challenge the government's governance. In May 2018, the Taliban banned house-to-house polio campaigns in the southern region but allowed site-to-site campaigns, which continued until early 2019. In April 2019, the Taliban banned polio campaigns throughout Afghanistan, suspended 1 national campaign and 2 subnational campaigns, and continued the suspensions until September 2019. Despite Taliban threats, the polio program vaccinated under-5s in relatively secure areas in August and September 2019, but could not reach >50% of the national targets [26].

The inaccessibility issue was resolved after the Taliban took control of the entire country. The number of inaccessible children was reduced from millions to zero in November 2021, the first campaign conducted fully under Taliban auspices. The number has remained zero in all campaigns conducted by June 2022 and beyond to date. The program succeeded in vaccinating 2.4 million children who in the 3 years before November 2021 had never been vaccinated. This is tremendous progress in the history of the GPEI. Inaccessibility is no longer a challenge for polio eradication at this time in Afghanistan.

The polio program targets approximately 10 million under-5s throughout the country each time. Prechange, the OPV coverage among target under-5s was 6 990 755 (69.9%) in November 2020 and 6 599 250 (71.2%) in January 2021. Coverage progressively increased to 8 556 783 (85.6%) in November 2021 and 10 061 780 (100.9%) in June 2022. This means that 3 462 530 more children were vaccinated in June 2022 than in January 2021. This is mainly thanks to reestablished access to children throughout Afghanistan. In addition, the total number of recorded missed children (including refusals, those not at home during the door-to-door visit, or asleep, neonatal, or sick) was also reduced from 140 140 (2.4%) in January 2021 to 131 202 (1.8%) in June 2022. The reduction of recorded missed children also indicates improvement in the quality and the coverage of target children in house-to-house polio campaign areas. Reductions in recorded missed children increase the likelihood of nationwide eradication of WPV1. Postchange access to all children in polio campaigns resulted in control of the 2020 to 2021 cVDPV2 outbreak, which paralyzed 308 and 43 children, respectively [5, 29].

The number of refusals among target children in house-to-house polio campaign areas was also progressively reduced from 57 530 (1.0%) in January 2021 to 40 140 (0.6%) in June 2022. Ranking after inaccessibility, OPV refusal is also a serious challenge to WPV1 eradication in Afghanistan, similar to several former polio-endemic countries’ experiences. Parents/caregivers might refuse OPV administration to their children on religious grounds (eg, belief that the vaccine contains haram [forbidden] ingredients such as pork, which a Muslim is not allowed to consume), campaign fatigue (frustration with multiple door knockings), other essential health and development services’ shortages in high-risk areas, deployment of nonlocal staff, young male volunteers’ presence, or shortage of female vaccinators/social mobilizers. The reduced rate of refusals may be due to the success of strategies adopted in 2020 and 2021 focusing on community engagement interventions across the country: women's inclusion in vaccination teams, use of mass and social media, and support from the Ministry of Haj and Auqaf (religious affairs), the Ministry of Education, and other ministries [27]. Polio communication interventions must intensify the polio campaign's efforts to promote and maintain vaccine acceptance and further convert refusals to acceptance by delivering other services addressing communities’ essential needs. Allocation of further resources in this area may expedite the interruption of WPV transmission in Afghanistan.

Postchange improvements in access also improved the AFP and environmental surveillance systems. AFP surveillance launched as part of the GPEI in 1997, met its quality indicators targets by 2003 [14], and is steadily improving, especially postchange. Its nonpolio AFP rate per 100 000 children aged <15 years (target ≥2) increased from 18.5 in 2020 to 24.3 in 2022 and 25.9 in 2023. Percentages of AFP cases with adequate specimens (target 80.0%) also improved, from 92.6% in 2020, to 94.4 in 2022 and 94.2 in 2023. Sewage sampling sites have nearly doubled, from 23 in 2020 to 38 in 2023 and 40 as of 31 January 2024. These findings are in line with the findings of the polio surveillance review conducted by 16 polio experts in mid-2022, who found that Afghanistan's surveillance system is achieving all sensitivity targets and the likelihood of undetected poliovirus transmission is low [30]. In addition, the MoPH is working with the WHO and the Bill & Melinda Gates Foundation to establish a polio laboratory, long desired by Afghanistan's government.

### Challenges to Polio Eradication

The gold standard and most accepted method of polio campaign implementation in low- and/or middle-income countries is the house-to-house visit. The increased number of target children in house-to-house visits from 5 283 681 in November 2021 to 7 180 055 in June 2022 and the decline in non–house-to-house interventions from 4 645 478 in November 2021 to 2 019 606 in June 2022 both signify expansion of the house-to-house modality in the eastern and southern regions with the highest concentration of zero-dose children [27]. While these are both good signs, OPV coverage in areas with nonhousehold modalities was 34.6% in November 2021 and 35.3% in June 2022, lower than the simultaneous OPV coverage in areas with house-to-house visits. This is a serious shortcoming that should be addressed.

There are various perceptions in banning house-to-house visits in some parts of the country. First, some policymakers think that when very essential and needed healthcare services are lacking, going house-to-house does not help the new government to “look good.” Second, some think that the mosque-to-mosque approach is popular and effective, and claims high coverage. Third, some think that security incidents (casualties) may harm the new authorities’ reputation in improving and maintaining peace and security [31].

In addition to the aforementioned challenges, the 131 202 missed children, including 40 140 refusals, recorded in June 2022, together with those unrecorded in the non–house-to-house areas throughout the country, pose challenges to national polio eradication. In addition, cross-border population movements, especially the forceful expulsion of 502 100 unregistered Afghan refugees from polio-endemic Pakistan between 15 September 2023 and 19 January 2024, and their settlement in polio-free areas of Afghanistan also pose a challenge to polio eradication [32]. GPEI and the donor community need to consider the essential health needs of the people and strengthen Afghanistan's healthcare system to a level where it can maintain its future polio-free status and pandemic/outbreak response. Advocacy efforts should be intensified to persuade Taliban representatives to allow house-to-house visits throughout Afghanistan. The major success in the most difficult place on the planet must not be jeopardized by possible international sanctions on the new regime.

### Limitations

The findings of this study should be interpreted in the light of the following limitations: First, polio campaign data are collected/compiled by thousands of vaccination teams with different education and knowledge levels, so are prone to some errors. However, polio campaign program staff receive training in each round, use unified data collection tools across the country, and have their operations monitored by supervisors, intracampaign monitors, coordinators, and provincial, regional, and national level staff. A quality-assurance field audit of administrative data is also carried out in each round.

Second, the polio program targets approximately 10 million children under 5 throughout the country, and this number serves as the denominator for the rates of vaccinated, inaccessible, and missed children and refusals. The same target estimates were used for the 2020–2022 polio campaigns. During the former government period, due to growing inaccessibility to children and population movements under conflict conditions, the program could not estimate or revise the target figure. Though it is therefore likely that recent rates are upper estimates, consistent improvement in the absolute number of vaccinated children and progressive decreases in the number of recorded missed children and refusals validate the relative accuracy of these estimates.

Third, in February 2022, there were attacks on the volunteers and the vaccination teams in the northern 2 provinces that resulted in the death of 8 vaccinators. The program therefore decided to forgo polio campaigns in the eastern and southern provinces to prevent possible casualties (unrelated to the extent of inaccessibility to children in these regions).

## CONCLUSIONS

Inaccessibility was a significant challenge to the GPEI in Afghanistan. More than half of WPV1 cases were reported from inaccessible areas. Despite nationwide disruptions in health services, the polio program has made significant progress postchange in Afghanistan. Inaccessibility to children is no longer a challenge to the GPEI in Afghanistan: the country has significantly improved OPV coverage and reduced the number of missed children and refusals during the 2022 polio campaigns. The program succeeded in interrupting 2020–2021 cVDPV2 transmission in the country. Its polio surveillance systems expanded and are less likely to miss any poliovirus circulation. Missed children and refusals and non–house-to-house approaches to polio campaign implementation caused 1 of 3 children to be missed in the past. The GPEI needs to benefit from the improved security situation, maintain the momentum by conducting quality SIAs, and intensify advocacy for house-to-house polio campaigns. The GPEI and its donors must meet the basic healthcare needs of this high-risk population and maintain and strengthen the healthcare system of Afghanistan as a prerequisite for achieving and maintaining the yet conceivable polio-free status of Afghanistan and the entire globe.

## Supplementary Data

Supplementary materials are available at *The Journal of Infectious Diseases* online (http://jid.oxfordjournals.org/). Supplementary materials consist of data provided by the author that are published to benefit the reader. The posted materials are not copyedited. The contents of all supplementary data are the sole responsibility of the authors. Questions or messages regarding errors should be addressed to the author.

## Supplementary Material

jiae129_Supplementary_Data

## Notes

***Disclaimer.*** In this article, “accessibility of under-five-year-old children to OPV vaccine after the political change in Afghanistan” refers to physical access of the polio workers to children in the country after the cessation of civil war, threats and direct attacks on polio workers, and other threats to physical security. The funder of this study had no role in the conception, design, data collection, data analysis and interpretation, or writing of the report.

***Financial support*.** This work was supported by the Bill & Melinda Gates Foundation (BMGF). General Incorporated Association LIAISON is funded by the BMGF to advocate for the Global Polio Eradication Initiative in Japan (INV-045916). This Article gives the views and opinions of the authors and is not endorsed by the Bill & Melinda Gates Foundation.
