# Correction to: Progress in Access and Oral Polio Vaccine Coverage Among Children Aged <5 Years in Polio Campaigns After the Political Change in Afghanistan

**DOI:** 10.1093/infdis/jiae334

**Published:** 2024-07-01

After the original publication of this article [Sabawoon W, Seino S, BM Pason et al. Progress in Access and Oral Polio Vaccine Coverage Among Children Aged <5 Years in Polio Campaigns After the Political Change in Afghanistan. *The Journal of Infectious Diseases*, April 2024, jiae129, https://doi.org/10.1093/infdis/jiae129] the authors requested to add necessary specifics to their disclaimer and financial support statements. The Disclaimer and Financial support sections in the version of this article currently available online have been updated to reflect the additional clarifications provided by the authors.

