# Supplementary material for: Progress in Access and Oral Polio Vaccine Coverage Among Children Aged <5 Years in Polio Campaigns After the Political Change in Afghanistan
Source: J Infect Dis. 2024 Apr 10;231(2):e438–45. doi: 10.1093/infdis/jiae129 (PMC11841624; doi:10.1093/infdis/jiae129)
Supplement: jiae129_Supplementary_Data [file jiae129_supplementary_data.docx]

**Supplementary annex**

**Detailed methods and background**

**Settings:** Afghanistan is a low-income country divided administratively into 34 provinces and 399 districts. Afghanistan’s polio eradication initiative (hereinafter "the polio program") operationally divides the country into 7 regions: Northern, Northeastern, Southern, Southeastern, Eastern, Western, and Central. United Nations estimates its population in 2021 at 39,835,000, of whom only 25·8% live in cities [33]. From November 2015, the polio program was part of a routine immunization program called the National Expanded Program on Immunization (NEPI). Under the NEPI, the government provides Bacillus Calmette-Guérin (BCG), hepatitis B birth dose, pentavalent vaccine (diphtheria, pertussis, tetanus, hepatitis B, and Haemophilus influenzae type b), Oral Polio Vaccine (OPV), Inactivated Polio Vaccine (IPV), 13-valent Pneumococcal Conjugate Vaccine (PCV), Measles-Rubella (MR) vaccine, and Rotavirus vaccine to children and Tetanus Toxoid (TT) vaccine to women of childbearing age.

The 12^th^ Independent Monitoring Board (IMB) meeting, a forum responsible for assessing progress in the detection and interruption of WPV’s spread globally, recommended that Afghanistan’s representatives visit the Emergency Operations Center (EOC) for polio eradication in Pakistan and assess its applicability to Afghanistan [34]. From November 2015, EOC development, based on lessons Pakistan had learned from Nigeria's experiences, sought to expedite the interruption of WPV transmission in Afghanistan. For better coordination and oversight, Afghanistan’s MoPH and partners established EOCs at the national level and in Eastern, Southern, and Western polio-high-risk regions [28].

Strong teams at the national and provincial levels which have maintained the program's neutrality were stymied by an inability to reach every child in Afghanistan, especially in the period from 2016 to August 2021 [14, 16-19]. In 2015 and 2016, despite worsening hostilities and insecurity across Afghanistan, the Government of Afghanistan escalated efforts to accelerate polio eradication [28]. Unique access-related challenges arose, including population pockets fully inaccessible for vaccination, partially accessible, or accessible with limitations in Taliban-controlled areas [16]. The situation further deteriorated from 2018 onwards: from May to December 2018, the Taliban banned house-to-house vaccination in most southern and southeastern provinces, rendering approximately one million children inaccessible. Although from January to April 2019 the Taliban permitted site-to-site programs in which children in designated community sites were vaccinated, such campaigns were again banned nationally by the Taliban beginning late April 2019 [17, 18].

These dynamic and constant features of inaccessibility, together with off-and-on bans on polio campaign implementation, deprived hundreds of thousands or millions of children of OPV and thwarted comprehensive nationwide polio campaigns. In March 2016, of 9,523,420 target children, 184,363 (1·9%) were inaccessible for vaccination [16]; the figure rose to 2,752,578 (28% of target children) in January 2020 and 3,381,642 (34%) in October 2020, when vaccinations resumed after five months of COVID-19-related suspensions; inaccessibility peaked at approximately 4,000,000 (40%) in March and June 2021 [18, 19]. Interruption of WPV1 transmission by the GPEI in such a context was impossible.

On August 15, 2021, the Islamic Republic of Afghanistan collapsed and the entire country came under the control of the Taliban (except for Panjshir province, which remained controlled by the Northern Alliance, one of the main power blocs in the Islamic Republic government, until it, too, came under full Taliban control on September 6, 2021). The new Taliban administration re-started polio campaigns in November 2021 throughout the country, including formerly inaccessible areas.

In the course of Supplementary Immunization Activities (SIAs), the polio campaign was implemented in two modalities: house-to-house (in areas formerly under control of the former government) and mosque-to-mosque (in areas formerly under predominantly Taliban control).

**Definitions**

***Target population***: The estimated number of children under five years of age (“under-fives”) eligible for OPV during the polio campaign.

***Under-fives’ OPV coverage:*** The percentage of target children who received OPV.

***Missed children:*** The number of under-fives who were recorded as not vaccinated for any reason in house-to-house campaign areas. The number of recorded missed children was divided by the number of house-to-house target under-fives multiplied by 100. Estimating the rate for the site-to-site and mosque-to-mosque modalities was impossible.

***Refusals:*** The number of under-fives in house-to-house areas who were missed because their parents refused to permit their children’s vaccination. The number of recorded missed children due to parents’ refusal, divided by the number of house-to-house target under-fives, multiplied by 100.

***Inaccessible area***: An area was considered “inaccessible” if there were overt or perceived threats of violence to polio vaccinators.

***Inaccessible children***: Target under-fives living in inaccessible areas during the polio campaign.

***Polio case from an inaccessible area***: A polio case that was reported from an inaccessible area and verified at their earliest convenience by the regional or national rapid response team responsible for detailed field investigations of new wild poliovirus (WPV) and Circulating Vaccine-Derived Poliovirus (cVDPV) cases.

***Campaign modality***: *House-to-house polio campaign* refers to a drive-in, also called the door-to-door approach, in which vaccinators go to each child's house and put drops in the children's mouths at the door. The vaccination status of all children in the house is recorded, the doors are marked, and the names of missed children are recorded and the house is revisited for vaccination of any missed children on another scheduled day of the campaign. Reasons for missing children are also recorded. In a *mosque-to-mosque polio campaign*, families are told to take children to the mosque for OPV vaccination. *Site-to-site polio campaigns* are an approach in which children are vaccinated at sites other than mosques. If the mosque is far from the children’s home, or the village is very densely populated, the vaccinator administers OPV in someone's guest house, in a public place, or anywhere that is appropriate for the vaccinator and the community. *Health-facility-to-health-facility* polio campaigns administer polio vaccine only in designated health facilities in areas under the control of the Taliban. In the non-house-to-house approaches, houses cannot be marked and the names of missed children cannot be recorded. The health-facility-to-health-facility approach might cover 3% to 20% of the target children in the Afghan context. The Taliban allowed this type of polio campaign implementation in September 2019 (see Figure 1). *Mixed modality* polio campaigns use various approaches that were permitted in specific geographic units. Figure 1 shows the distribution of campaign implementation modality by access status of provinces.

***Annualized non-polio Acute Flaccid Paralysis (AFP) rate:*** At least one case of non-polio AFP should be detected annually per 100,000 population aged less than 15 years. In endemic regions, to ensure even higher sensitivity, this rate should be two per 100,000. The indicator shows the sensitivity of the AFP surveillance system [35].

***Stool adequacy rate***: All AFP cases should have a full clinical and virological investigation with at least 80% of AFP cases having ‘adequate’ stool specimens collected. ‘Adequate’ stool specimens are two stool specimens of sufficient quantity for laboratory analysis, collected at least 24 hours apart, within 14 days after the onset of paralysis, and arriving in the laboratory by reverse cold chain and with proper documentation. This indicator shows the completeness of the case investigation [35].

***Political change:*** The collapse of the Islamic Republic of Afghanistan and its replacement by the Taliban, the Islamic Emirate of Afghanistan, in August 2021, following the signing of an agreement between the United States and the Taliban in February 2020 on the withdrawal of international forces from Afghanistan by May 2021 (in actuality completed in August 2021) [36].

**Data collection and analysis**

***Data source:*** National Emergency Operations Center (NEOC), Ministry of Public Health, Afghanistan, WHO's Regional Office for the Eastern Mediterranean (EMRO), and GPEI websites.

***Data collection:*** We obtained data from the NEOC of MoPH, Afghanistan, downloaded from the WHO and GPEI websites. The source for the AFP, WPV1, and cVDPV2 cases is the acute flaccid paralysis (AFP) surveillance data for the years 2015–2023, regularly collected by the World Health Organization (WHO). The AFP surveillance of the country is sensitive, less likely to miss any polio case, and available in both accessible and inaccessible areas of the country [14]. The OPV coverage and missed-children-related data came from Supplementary Immunization Activities (SIAs), the polio campaign's administrative data for the years 2020 to 2022. The data are collected by the vaccinators from each house, mosque, or site using specific data-collection tools. The data are compiled at team, cluster, district, province, region, and national levels.

***Data analysis:*** In this report, we analyzed the national-level data. First, we described the trend in the number of AFP, WPV1, and cVDPV2 cases and in the proportion of WPV1 cases reported from inaccessible areas to confirm the effects of inaccessibility on polio eradication. Second, we described trends in the numbers and rates of vaccinated, inaccessible, and missed children and refusals in polio campaigns conducted post-change to visualize progress achieved by the polio campaigns conducted pre-change, November 2020 and January 2021. Third, we described the trends in the annualized non-polio AFP rate per 100,000 children under fifteen years old and the percentage of adequate stool specimens. Fourth, we described trends in the numbers and rates of children vaccinated by the various modalities of the SIAs’ implementation.

***Role of the funding source:*** The funder of this study had no role in the conception, design, data collection, data analysis and interpretation, or writing of the report.

**Table 3: Impacts of warfare - inaccessibility, bans, conflict conditions, and implementation modality of polio campaigns, 2018-2021, by month**

| **Month** | **Year** | | | |
| --- | --- | --- | --- | --- |
|  | **2018** | **2019** | **2020** | **2021** |
| **January** | No ban | H2H is banned but S2S permitted in high-risk provinces | Millions of children were inaccessible on NIDs | Levels of inaccessibility worsened |
| **February** | No ban | H2H is banned but S2S permitted in high-risk provinces | SNIDs left millions of children inaccessible | SNIDs were planned, but postponed due to intensified warfare |
| **March** | No ban | H2H is banned but S2S permitted in high-risk provinces | NIDs paused due to the COVID-19 pandemic | NIDs planned, but postponed due to intensified warfare |
| **April** | No ban | H2H is banned but S2S permitted in high-risk provinces | NIDs paused due to the COVID-19 pandemic | NA |
| **May** | H2H ban in high-risk provinces | Complete ban by the Taliban in the entire country | NIDs paused due to the COVID-19 pandemic | NIDs planned, but postponed due to intensified warfare |
| **June** | H2H ban in high-risk provinces | Complete ban by the Taliban in the entire country | NIDs paused due to the COVID-19 pandemic | NA |
| **July** | H2H ban in high-risk provinces | Complete ban by the Taliban in the entire country | NIDs paused due to the COVID-19 pandemic | NIDs planned, but postponed due to intensified warfare |
| **August** | H2H ban in high-risk provinces | Complete nationwide ban by the Taliban, but the polio program was gradually resumed by the Government in accessible areas | NA | Collapse of the Afghan government, regime change |
| **September** | H2H ban in high-risk provinces | At the end of the month, the Taliban lifted the ban partially and allowed HF2HF polio campaign activities in their areas | SNIDs implemented | NIDs planned, but postponed due to the recent political transition |
|  | NA | NA | Complementary immunization resumed. On the Torkham border with Pakistan, all-age OPV vaccination started | NA |
| **October** | H2H ban in high-risk provinces | HF2HF campaign permitted in high-risk provinces | Millions of children were left inaccessible on NIDs | NA |
|  | H2H modality covered 57% of target children in accessible areas | NA | NA | NA |
| **November** | H2H ban in high-risk provinces | HF2HF campaign permitted in high-risk provinces | Millions of children were left inaccessible on NIDs | The first nationwide NIDs were conducted in the entire country |
|  | NA | H2H modality covered 64% of target children in accessible areas | NA | NA |
| **December** | H2H ban in high-risk provinces | HF2HF campaign permitted in high-risk provinces | NA | The second nationwide NIDs were conducted in the entire country |
|  | H2H modality covered 68% of target children in accessible areas | NA | NA | NA |

**Figure: Inaccessibility and implementation modality of supplementary immunization activities by district, September–November 2020**

^
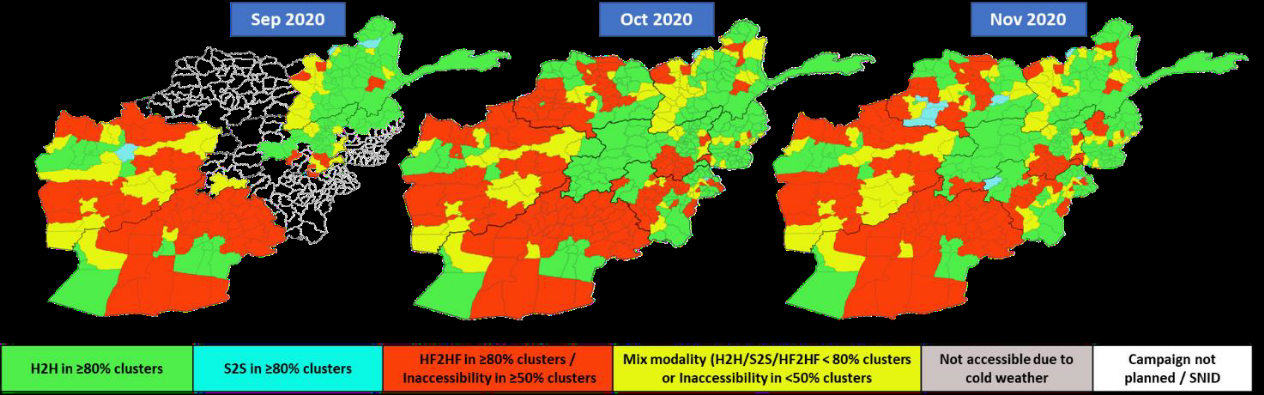
Adopted from National Emergency Action Plan 2021 [37]^

^NIDs: National Immunization Days, a massive national-level polio campaign; SNID: sub-National Immunization Days, a campaign in high-risk provinces; H2H: house-to-house polio campaign; S2S: site-to-site polio campaign; HF2HF: health facility-to health facility polio campaign; Cluster: an area where polio operations are covered by polio vaccination teams.^

**References**

1. Simpson DM, Sadr-Azodi N, Mashal T, et al. Polio eradication initiative in Afghanistan, 1997-2013. J Infect Dis 2014 Nov 1;210 Suppl 1:S162-72. DOI: 10.1093/infdis/jiu022.
2. Martinez M, Shukla H, Nikulin J, et al. Progress toward poliomyelitis eradication—Afghanistan, January 2016–June 2017. MMWR Morb Mortal Wkly Rep 2017;66:854–8. PMID:28817551 https://doi. org/10.15585/mmwr.mm6632a5.
3. Martinez M, Shukla H, Nikulin J, Mbaeyi C, Jorba J, Ehrhardt D. Progress toward poliomyelitis eradication—Afghanistan, January 2018–May 2019. MMWR Morb Mortal Wkly Rep 2019;68:729–33. PMID:31437144 https://doi.org/10.15585/mmwr.mm6833a4.
4. Martinez M, Akbar IE, Wadood MZ, Shukla H, Jorba J, Ehrhardt D. Progress toward poliomyelitis eradication—Afghanistan, January 2019–July 2020. MMWR Morb Mortal Wkly Rep 2020;69:1464–8. PMID:33031360 https://doi.org/10.15585/mmwr.mm6940a3.
5. Sadegh KS, Akbar IE, Wadood MZ, Shukla M, Jorba J, Chaudhury S, Martinez M. Progress Toward Poliomyelitis Eradication — Afghanistan, January 2020–November 2021. MMWR Morb Mortal Wkly Rep 2022;71:86-89 DOI: 10.15585/mmwr.mm7103a3.
6. National Emergency Operational Center. Polio update: Afghanistan. As on July 15, 2015. http://polioeradication.org/wp-content/uploads/2016/08/4.1_14IMB.pdf (accessed July 24, 2020).
7. UN data. Afghanistan. General Information. Afghanistan Population 2021. UNData app (accessed June 30, 20220).
8. Independent Monitoring Board. Global Polio Eradication Initiatives. Now is the time for peak performance. IMB 12th report: October 2015. http://polioeradication.org/wp-content/uploads/2016/0701.pdf (accessed July 23, 2020).
9. Global Polio Eradication Initiatives. Surveillance indicators. Available at: https://polioeradication.org/polio-today/polio-now/surveillance-indicators/ (accessed February 1, 2024).
10. Marty FJ. The US -Taliban deal: a year later. The Diplomat. February 2021. Issue 75. Available at: https://thediplomat.com/2021/01/the-us-taliban-deal-a-year-later/ Accessed July 19, 2022.
11. National Emergency Action Plan. Polio Eradication Initiative, Afghanistan. 2021. <https://polioeradication.org/wp-content/uploads/2022/02/3.1-Afghanistan-NEAP-for-Polio-2021.pdf> (accessed February 15, 2023).
